# Mechano-Chemical Effect of Gelatin- and HA-Based Hydrogels on Human Retinal Progenitor Cells

**DOI:** 10.3390/gels9010058

**Published:** 2023-01-11

**Authors:** Pierre C. Dromel, Deepti Singh, Alfredo Alexander-Katz, Motoichi Kurisawa, Myron Spector, Michael Young

**Affiliations:** 1 Schepens Eye Research Institute of Massachusetts Ear and Eye, Mass General Brigham, Harvard Medical School, 20 Staniford Street, Boston, MA 02144, USA; 2 Department of Materials Science and Engineering, Massachusetts Institute of Technology, 77 Massachusetts Ave., Cambridge, MA 02139, USA; 3 Japan Advanced Institute of Science and Technology, 1-1 Asahidai, Nomi 923-1292, Ishikawa, Japan; 4 VA Boston Healthcare System, Brigham and Women’s Hospital, Harvard Medical School, Boston, MA 02155, USA

**Keywords:** retinal regeneration, biomaterials, hydrogel, stiffness, cellular differentiation

## Abstract

Engineering matrices for cell therapy requires design criteria that include the ability of these materials to support, protect and enhance cellular behavior in vivo. The chemical and mechanical formulation of the biomaterials can influence not only target cell phenotype but also cellular differentiation. In this study, we have demonstrated the effect of a gelatin (Gtn)—hyaluronic acid (HA) hydrogel on human retinal progenitor cells (hRPCs) and show that by altering the mechanical properties of the materials, cellular behavior is altered as well. We have created an interpenetrating network polymer capable of encapsulating hRPCs. By manipulating the stiffness of the hydrogel, the differentiation potential of the hRPCs was controlled. Interpenetrating network 75 (IPN 75; 75% HA) allowed higher expression of rod photoreceptor markers, whereas cone photoreceptor marker expression was found to be higher in IPN 50. In vivo testing of these living matrices performed in Long–Evans rats showed higher levels of rod photoreceptor marker expression when IPN 75 was injected versus IPN 50. These biomaterials mimic biological cues that are required to simulate the dynamic complexity of natural retinal ECM. These hydrogels can be used as a vehicle for cell delivery in vivo as well as for expansion and differentiation in an in vitro 3D system in a highly reproducible manner.

## 1. Introduction

Retinal degenerations such as retinitis pigmentosa and age-related macular degeneration result in irreversible loss of vision. Due to the lack of self-repair ability of the mammalian retina, replacing lost photoreceptors is a strategy being developed to treat these blinding diseases. Numerous cell therapy techniques have been explored to regenerate or replace affected tissue or cells [1]. Neuroprotective treatments can in theory preserve vision but require surviving host photoreceptor cells for the therapy to be successful [2,3]. For cell replacement to become a viable treatment for retinitis pigmentosa clinically, functionally competent neural retinal cells in therapeutically relevant quantities are required. To achieve this goal, human retinal progenitor cells (hRPCs) have been explored. These cells have shown the ability to survive, differentiate and engraft into the host retina, where they promote host photoreceptor rescue, improving visual function in animals [2]. Failure of hRPCs in multi-center phase 2 clinical trials raised the question of the suitability of the delivery vehicle, phosphate buffered saline (PBS).

Cell death during transplantation is largely attributed to the low viability of the cells themselves, but we have found that it is exacerbated by the lack of a suitable carrier capable of forming a protective envelope around the cells [4]. Most retinal stem or progenitor cells, when delivered using conventional injection techniques employing PBS, show low viability and poor integration with the host tissue [5]. An ideal polymer would enhance cell survival and degrade within a desired time frame without triggering an inflammatory response from the host [6]. It is clear the environment at the degenerative site in the outer retina is highly unfavorable for the survival of grafted cells [7]. Without structural support within the retina, transplanted cells lack cell–cell and cell–substrate support and therefore undergo apoptosis in large numbers. The cells that survive lack a matrix to be spatially retained and typically leave the injection site for the surrounding viable host tissue [8]. It is critical to identify a suitable scaffold for retinal cell delivery that promotes regeneration. Many biomaterials have been shown to play major roles in maintaining cell phenotype, proliferation, and differentiation [4,9]. Other studies have shown that material properties such as stiffness can influence and even direct stem cell differentiation [1]. However, another critical attribute that merits examination is the difference between the in vitro culture conditions and in vivo systems. The cells cultured in the lab are provided with ideal growth conditions to achieve the highest viability, contrasting to challenging degenerative in vivo conditions. Biomaterials can play a critical role in controlling the in vivo conditions cells are exposed to during and after transplantation. Cells encapsulated within the gels are provided a 3D protective microenvironment preventing direct exposure to the hostile host tissue conditions.

**Figure 1:**
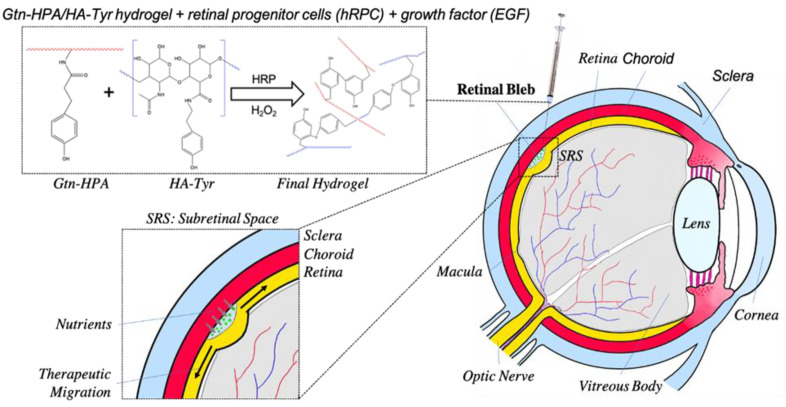
Schematic of the proposed therapeutic approach using hydrogels for retinal regeneration. Retinal progenitor cells (hRPCs) and growth factor (EGF) are mixed with liquid solutions of Gtn-HPA and HA-Tyr. As soon as the catalyst (HRP) and crosslinker (H_2_O_2_) are added, the final therapeutic is injected into the subretinal space through the sclera and the retina with a 31-gauge needle. This creates a retinal bleb, which contains the final solid hydrogel encapsulating cells and growth factors. This therapeutic then migrates along the retina, releasing cells to engraft in the host tissue.

The principal objective of this study was to test an in situ crosslinked hydrogel and analyze its effect on in vivo transplantation of hRPCs into the subretinal space of-Long–Evans rats over time. We formulated Gtn-HPA and HA-Tyr hydrogels with a gelation time of 30 to 180 s. One aim of this study was to explore the use of a hydrogel as a “protective envelope” for cells that experience high shear stress during injection. We performed in vivo xeno-transplantation of hRPCs in PBS, Gtn-HPA, and IPN75 into the subretinal space of immunosuppressed rats of the Long–Evans strain, as seen in Figure 1. Injected retinas were analyzed 3 days and 3 weeks post-surgery for the survival, engraftment, and viability of hRPCs.

## 2. Results

### 2.1. Testing of Short-Term Cell Viability and Phenotype in Gtn-HPA and HA-Tyr Hydrogels

**Figure 2:**
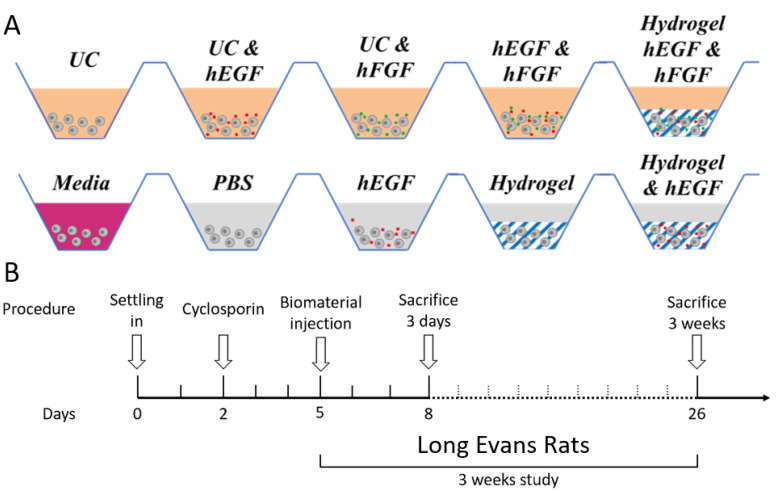
Experimental design to test the effect of Gtn-HPA and HA-Tyr on hRPCs. (**A**) hRPCs were cultured for 14 days in different conditions: in PBS, Ultraculture base medium (UC), in media including growth factors, with hEGF, with hFGF, in hydrogels. hRPCs cultured for 14 days in different media and tissue culture were then analyzed for their phenotype with flow cytometry and immunocytochemistry. (**B**) Encapsulated hRPCs were injected in the subretinal space of Long–Evans rats for a 3-week study.

We tested the immediate impact of hydrogels on cell viability and phenotype at 1 day in culture (Figure 2). This helps understand and quantify the impact of the crosslinking and seeding process on hRPC viability.

**Figure 3:**
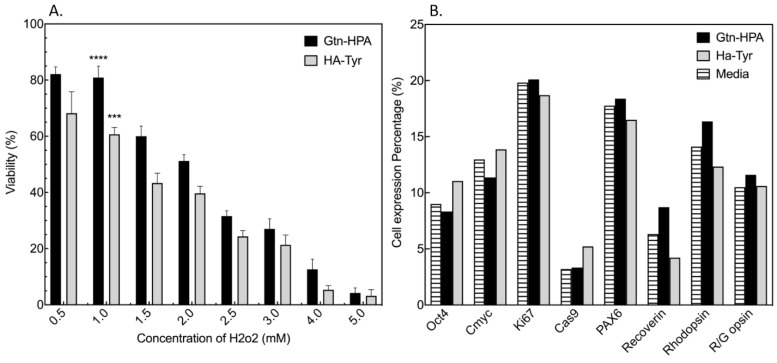
Viability and phenotype assay on hRPCs encapsulated in Gtn-HPA/HA-Tyr after 1 day. (**A**) Viability was measured by counting the number of live and dead cells in the viability assay performed with CalceinAM and Ethidium Bromide. Data of each group were calculated from 15 randomly chosen fields in each group using confocal fluorescence microscopy. Viability was observed to be significantly decreasing with the increase in hydrogen peroxide concentration. **** *p* = 0.0001; *** *p* = 0.001. (**B**) hRPCs cultured with media or in Gtn-HPA were analyzed for their phenotype with flow cytometry. No significant difference was observed at short time between both groups for all retinal, stemness, rods, cones, proliferation and apoptosis markers.

One potential stressor in cells encapsulated in Gtn-HPA and HA-Tyr is oxidative stress due to the presence of hydrogen peroxide as a crosslinker (usually cytotoxic to cells in high doses). We performed a viability assay on cells encapsulated in hydrogels with increasing concentration of H_2_O_2_ (with constant HRP at 0.1 U/mL) to find the optimal formulation for both homopolymeric networks: this could then be transferred to IPN (Figure 3A). To accurately quantify the number of viable cells, a live/dead assay was performed. For both samples (Gtn-HPA and HA-Tyr) crosslinking with H_2_O_2_ at 1 mM seemed to provide the highest biocompatibility with viability ranging from 60% in HA-Tyr to 80% for Gtn-HPA. We observed at low concentration of H_2_O_2_ (<0.8 mM) almost no gel formation, with higher viability at 0.5 mM. This is likely because in this case H_2_O_2_ present is not sufficient to form a gel and therefore cells are in a 2D formation. Therefore, we increased hydrogen peroxide concentration up to 5 mM to look for its cytotoxic effect on cells. Oxidative stress was already high at 2.5 mM, with a viability ranging from 20% to 35%, while being minimal at 5 mM, where most cells died (only 5–8% viable). This broad testing of hydrogen peroxide effect on cell viability in both homopolymeric networks suggested that a concentration of 1 mM should be used for further studies.

The second important impact of hydrogels on cell fate in this H_2_O_2_ study relates to hRPC differentiation into different retinal lineages as matrix is known to influence the cellular behavior [10]. We analyzed this effect with a phenotype assay, measuring the expression of common retinal and other markers (stemness, proliferation, apoptosis, retinal, cone and rod) for hRPCs in their normal condition (in 2D culture with media) and encapsulated in Gtn-HPA hydrogels for 1 day. As seen in Figure 3B, no significant difference was found for any of the markers within the group at this time point. This result suggests that hRPCs maintain their phenotype when encapsulated in Gtn-HPA and HA-Tyr for short time points. This short-term phenotype analysis enables us to have a basis on hRPCs retinal marker expression both in a 2D and 3D environment and allows us to compare to previous work [11,12].

### 2.2. Improvement of Viability and Proliferation of hRPCs Encapsulated in Biocompatible Hydrogels

**Figure 4:**
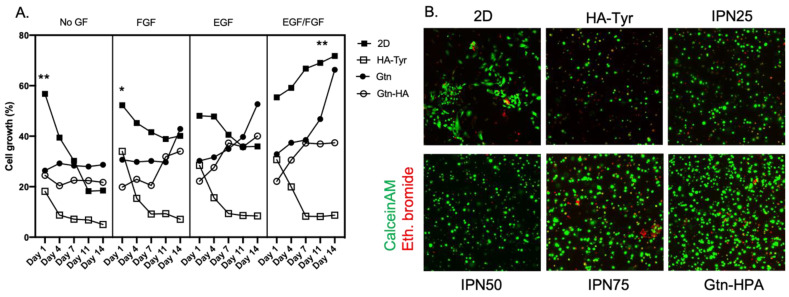
Viability and proliferation assay for hRPCs encapsulated in IPNs. (**A**) Cell growth was obtained with Alamar Blue staining for *n* = 5 replicates using colorimetry. Cells were found to possess a significantly higher growth when encapsulated in pure Gtn-HPA or with media. (* *p* < 0.05, ** *p* < 0.005). (**B**) Live/dead staining of hRPCs in different tissue culture conditions. Gtn-HPA shows the highest number of live cells while HA-Tyr shows highest number of dead cells.

Cell growth and viability were measured with Alamar Blue, CalceinAM and Ethidium Bromide, as explained in methods, at day 1, 4, 7, 11 and 14. Figure 4A shows the hRPCs growth in different hydrogels and with different nutrients over time. Deprived of any growth factor (no GF) cells tend to have a significantly less expansion when encapsulated in hydrogels, especially in pure HA-Tyr, compared to culture in 2D (positive control) or in hydrogels with higher gelatin content. By adding either EGF or FGF as discussed in Figure 2 to the hydrogels more cell growth was observed for groups containing a high content of gelatin and 2D culture. Furthermore, by adding both FGF and EGF at concentrations present in the defined media significantly higher cell growth was observed for cells cultured in 2D or in pure Gtn-HPA even after 14 days compared to all other groups. Overall, when depriving hRPCs from growth factor a visible decreasing cell growth was observed over time (from day 1 to day 14) but when including both growth factors cells were found to be proliferating at a high rate, increasing almost exponentially for hRPCs encapsulated in pure Gtn-HPA (Figure 4B).

To further confirm these results, we performed a live/dead staining assay by adding CalceinAM (FITC in green) and Ethidium Bromide (APC in red) to hRPCs in culture, as seen in Figure 4B. Live cells are shown in green and dead cells in red. Fifteen randomly chosen fields were chosen in each group to calculate viability data. Cells were seeded in all samples at the same concentration, enabling for a correct measurement of the viability in these different hydrogels. As we have seen with the cell growth experiment, cells tend to be more proliferative and viable when both EGF and FGF are added. Therefore, we present here only this nutrients groups with different tissue culture and hydrogels: 2D culture, HA-Tyr, IPN25, IPN50, IPN75 and Gtn-HPA. As seen in Figure 4B, hRPC viability increases significantly with the amount of gelatin in the hydrogel mix, reaching the highest viability and highest number of live cells when cells are encapsulated in pure Gtn-HPA.

Overall, these results suggest that adding both EGF and FGF into the hydrogels can greatly enhance the proliferation capabilities and viability of hRPCs in vitro (Figure 4A). This suggests a specific advantage of using our hydrogels as a delivery vehicle for retinal regeneration as it enables the encapsulation of not only cells but also growth factors and other nutrients to improve the viability of cells in vivo and therefore their potential engraftment and survival long term, thereby improving retinal regeneration.

### 2.3. Encapsulated hRPC Differentiation Is Driven by the Interpenetrating Network Content

One objective of this experiment is to examine the effect of hydrogels based on gelatin and hyaluronic on the phenotype and differentiation of hRPCs after 14 days in culture. To address this, we performed a phenotype analysis with flow cytometry on hRPCs encapsulated in different hydrogels at day 1, 4, 7, 11 and 14. This phenotype analysis was performed on markers of interest: stemness, retinal, photoreceptor, cone and rod as hRPCs being derived from fetal retina can only differentiate into retinal cells. Our approach was to analyze phenotypic differences in photoreceptors population. The flow cytometry analysis was performed with the Miltenyi MACSQuant flow cytometer. Figure 3B show the result of the phenotype analysis for Oct4-stemness, PAX6-retinal, recoverin-photoreceptor, rhodopsin-rods and R/G opsin-cones. For all markers, the same gating strategy (population of event or cells to be considered as positive) was applied: gating the cell population (FSC-A vs. SSC-A), gating the single-cell population (FSC-A vs. FSC-H), and then gating the DAPI-positive population (VioBlue-A vs. FSC-A). This single-cell-DAPI-positive gate correspond to the population of cells we compare for all markers (retinal, stemness, photoreceptor, rods, and cones). The data presented in the following figures show days 4, 7, 11 and 14 as data in day 1 was not relevant due to the short-term culture of cells in hydrogels. Cells usually need 4–7 days to express a different phenotype due to their response to different environments.

**Figure 5:**
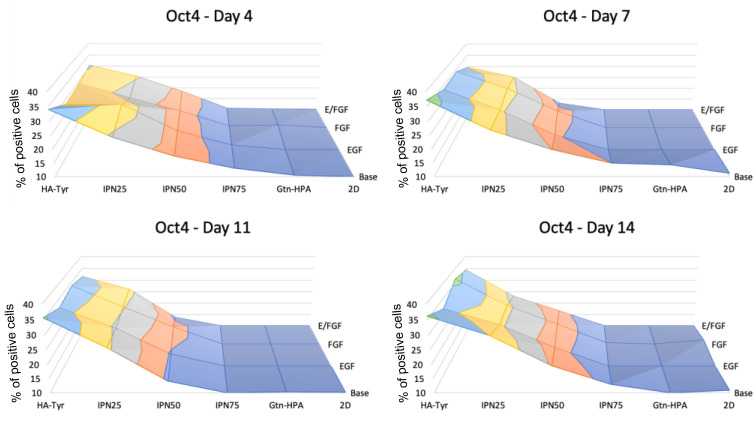
Stemness expression (Oct4) in hRPCs in different tissue culture. Cells were cultured for 2 weeks and phenotype was analyzed at day 4, 7, 11, and 14 for *n* = 5 replicates with flow cytometry. Map surfaces show the medium component on the *x*-axis (base medium, EGF, FGF, or both), the tissue culture on the *y*-axis (2D, Gtn-HPA, IPN75, IPN50, IPN25, and HA-Tyr) and the expression of Oct4 is shown on the *z*-axis.

Figure 5 shows the expression of Oct4, a key gene that regulate stemness in progenitor cells [13]. The expression of Oct4 was observed to be similar from day 4 to day 14 showing a significant increase with the addition of HA in the tissue culture, being the highest for pure HA-Tyr with 35% compared to Gtn-HPA or 2D with less than 12%. The change in nutrients did not impact the stemness of hRPCs which showed similar expression for all sample. This result can be compared to many studies that have shown the importance and effective association of stiffness of biomaterials on stem cell culture [14,15]. This results further corroborates that by increasing the gelatin content of hydrogels, one can increase hRPC differentiation towards a specific retinal cell fate.

**Figure 6:**
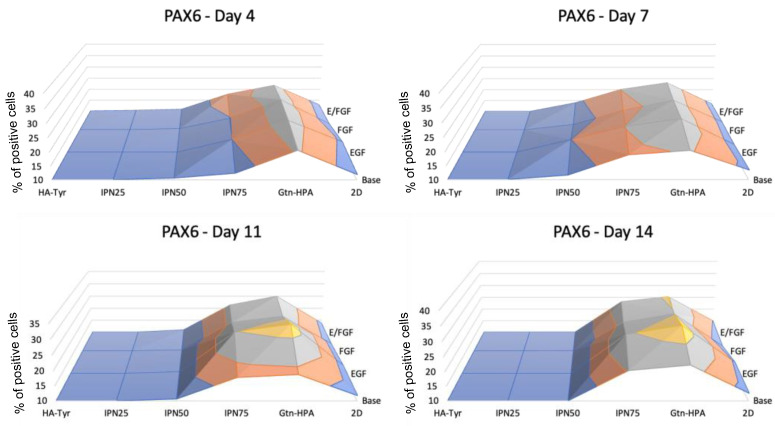
Phenotype assay for retinal marker PAX6 of hRPCs in different tissue culture. Cells were cultured for 2 weeks and phenotype was analyzed at day 4, 7, 11, and 14 for *n* = 5 replicates with flow cytometry. Map surfaces show the medium component on the *x*-axis (base medium, EGF, FGF, or both), the tissue culture on the *y*-axis (2D, Gtn-HPA, IPN75, IPN50, IPN25, and HA-Tyr) and the expression of PAX6 is shown on the *z*-axis.

A second marker which was analyzed is PAX6, as shown in Figure 6. PAX6 is an early retinal cell marker [16]. The exact opposite trend was observed, showing a significantly higher expression of PAX6 for cells cultured in Gtn-HPA and high-gelatin-content hydrogels. The expression of PAX6 was seen to increase with time, reaching a plateau after 10 days. Gtn-HPA and IPN75 show the highest expression with 30% and 28%, respectively, after 11 days compared to less than 10% for cells cultured in HA-Tyr. A similar trend as for Oct4 was also observed for the nutrient effect on PAX6 expression. The data suggest that there is no impact from EGF or FGF on the early retinal marker expression of hRPCs. This early retinal marker expression difference suggests that hRPC differentiation could be driven towards retinal cells by using tissue culture with high gelatin content and lower stiffness.

**Figure 7:**
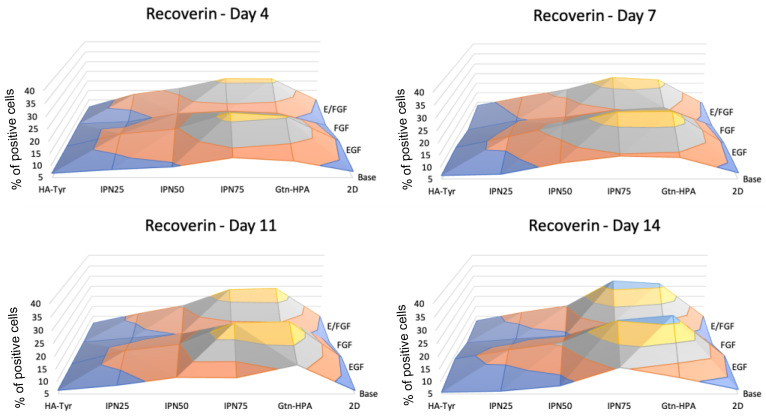
Photoreceptor expression (recoverin) in hRPCs cultured in hydrogels. Cells were cultured for 2 weeks and phenotype was analyzed at day 4, 7, 11, and 14 for *n* = 5 replicates with flow cytometry. Map surfaces show the medium component on the *x*-axis (base medium, EGF, FGF, or both), the tissue culture on the *y*-axis (2D, Gtn-HPA, IPN75, IPN50, IPN25, and HA-Tyr) and the expression of recoverin is shown on the *z*-axis.

As hRPCs are typically designed to be injected in the subretinal space to replace dead photoreceptors, we analyzed a specific photoreceptor marker: recoverin. This marker is expressed by both cones and rods with no specificity. As seen in Figure 7, a significant increase in recoverin expression was found from day 4 to day 14. Moreover, a similar trend as for PAX6 was observed with the highest expression for Gtn-HPA and IPN75 after 14 days: 36% and 34%, respectively. Of note is that the marker was strongly impacted by the different nutrients added to hRPCs. All samples which did not receive EGF (base medium and FGF only) were found to be significantly lower than other samples. Therefore, both EGF and EGF/FGF samples show higher expression.

**Figure 8:**
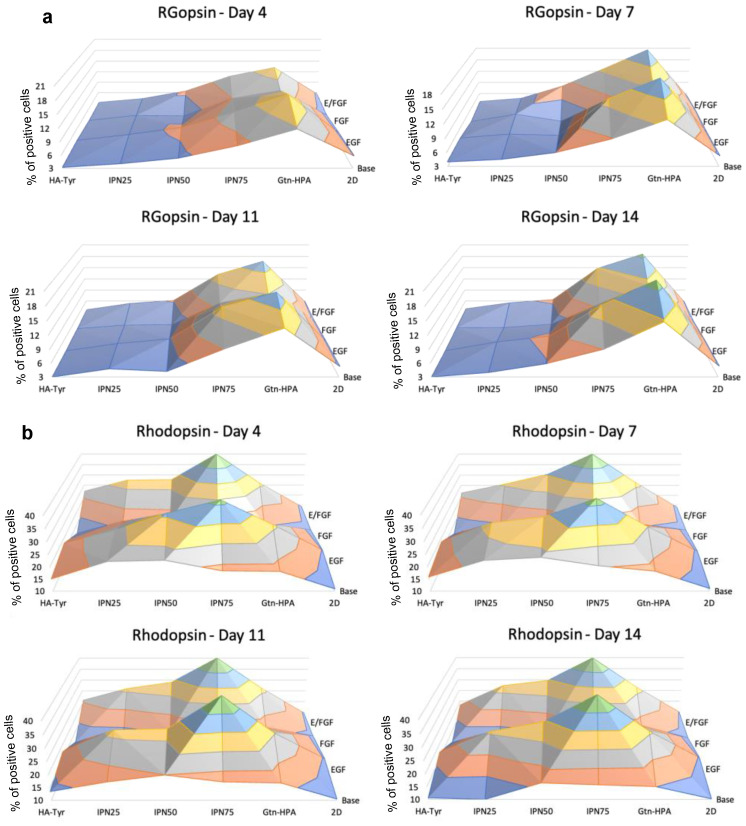
Phenotype assay for rod (rhodopsin) and cone (opsin) markers of hRPCs. Cells were cultured for 2 weeks and phenotype was analyzed at day 4, 7, 11, and 14 for *n* = 5 replicates with flow cytometry. Map surfaces show the medium component on the *x*-axis (base medium, EGF, FGF, or both), the tissue culture on the *y*-axis (2D, Gtn-HPA, IPN75, IPN50, IPN25, and HA-Tyr) and the expression of (**a**). R/G-opsin (**b**). Rhodopsin is shown on the *z*-axis. Cones show a specific peak for Gtn-HPA while rods show a peak for IPN75.

Next, we analyzed both a rod (rhodopsin) and cone marker (RGopsin). The data in Figure 8 show the same trend as for recoverin with a higher expression for cells cultured in high-gelatin-content hydrogels, with an increasing expression from day 4 to day 14. The same trend for FGF was observed for both rod and cone markers, strongly suggesting that the addition of EGF in the culture of hRPCs significantly increase their differentiation towards photoreceptors. A promising result was found when analyzing rod and cone markers: hRPCs have a higher differentiation towards rods for IPN75 while having a higher differentiation towards cones for Gtn-HPA.

Of note is that the expression of all markers was found to be significantly lower for cells cultured in typical 2D tissue protocol compared to encapsulation in hydrogels, suggesting that hydrogels can greatly impact the differentiation of hRPCs. Overall, this phenotype analysis of hRPCs encapsulated in different hydrogels with different nutrients added shows a differentiation towards a specific type of retinal cells by modulating the stiffness and nutrients added to hRPCs. We showed that stemness expression greatly increases with the addition of HA while early retinal marker decreases. Photoreceptor, rod, and cone markers were found to be significantly higher for cells that received EGF. Rod marker expression was found to be the highest in IPN75 and cone marker expression for Gtn-HPA. Finally, Alamar Blue and viability enabled us to confirm that hydrogels with a high HA content did not enhance cell attachment, proliferation, and viability.

### 2.4. Subretinal Transplantation of Encapsulated hRPCs in Different Hydrogels

This longest time point tested (3 weeks) enabled the study of survival of transplanted hRPCs (in PBS, Gtn-HPA, or IPN75). hRPC engraftment was evaluated using immunohistochemistry for every 4th or 6th section of each eye of each group. Sections were triple stained, as previously described, with DAPI-VioBlue (nuclei stain), STEM121-FITC (human marker), and either rhodopsin-APC (rod marker) or RGopsin (cone marker). These different stains enable us to analyze not only the engraftment of hRPCs (by using the human marker) but also their differentiation. The different injection groups were hRPCs in PBS, in Gtn-HPA, in Gtn-HPA with hEGF, in IPN75 or in IPN75 with hEGF. These groups were chosen due to the result seen with the in vitro phenotype assay. By analyzing the result of this in vivo injection at 3 weeks, we were able to measure the effect of stiffness (due to different biomaterials) and nutrients.

**Figure 9:**
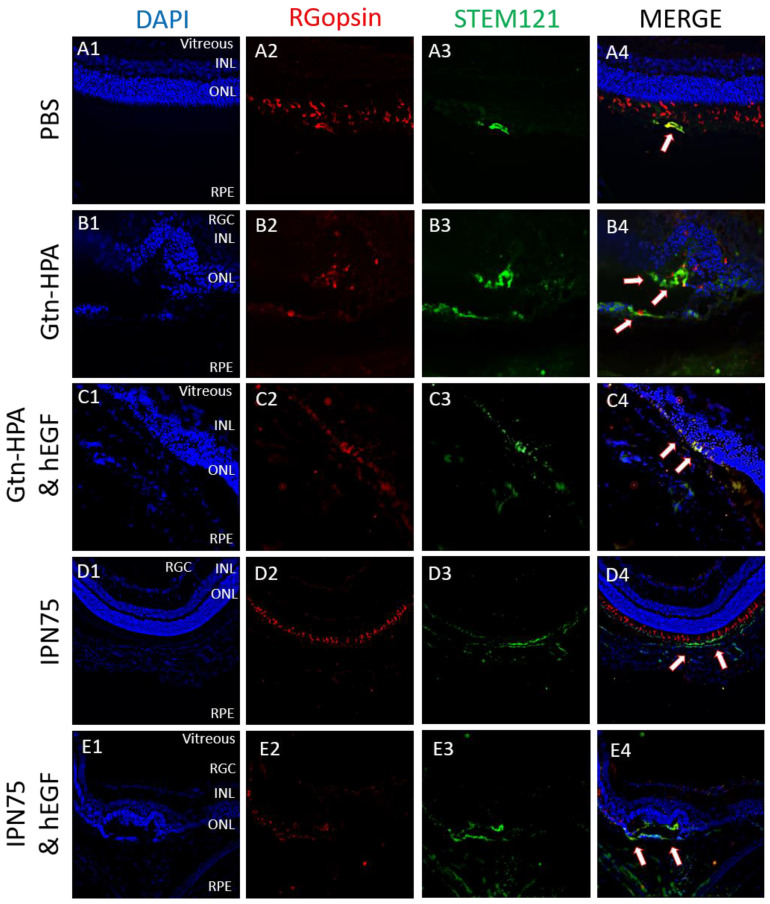
The 3-week transplantation of hRPC in IPN and PBS. hRPCs survived in immunosuppressed rats for 3 weeks following subretinal injection. All images were taken at 40X magnification. (**A2**–**E2**) are different test conditions stained with RG-opsin (red); (**A3**–**E3**) show STEM121 (green) staining. Nuclei are counterstained with DAPI in (**A1**–**E1**) and the last column (**A4**–**E4**) shows the merge overlay image. Red arrows show engrafted hRPCs aligned with DAPI near the ONL layer. Scale bar: 200 µm.

Upon triple staining of the retina, in all groups, we observed hRPCs in the subretinal space of Long–Evans rats with distinct morphology and phenotypic expression. Cells were analyzed for DAPI, human marker and cone marker to measure overall engraftment (Figure 9). Specifically, cells injected in PBS showed poor engraftment with a low number of cells expressing STEM121 and being clustered at the injection site with low migration, as seen in Figure 9A. Cells encapsulated in Gtn-HPA and IPN75 show high survival and engraftment in the subretinal space. These cells have a morphology similar to native photoreceptors and have a higher surface coverage, showing a higher migration from the injection site (Figure 9B,D). This suggests that encapsulating hRPCs in hydrogel could enhance the regeneration by increasing the migration of cells. The addition of hEGF showed little to no significance in the overall engraftment of hRPCs. However, as seen in Figure 9C,E, we observed that hEGF increased the presence of double-stained cells (expressing both cone and human markers (red arrows)). This result is in agreement with our in vitro experiment, showing that hEGF does not impact the viability of hRPCs but can drive their differentiation in vitro and in vivo.

These results confirm our findings for the short-term in vivo experiments: hRPCs encapsulated in gel show higher engraftment than those injected in PBS. However, due to the result of the in vitro data, we also analyzed the specific expression of injected cells with two specific markers: rhodopsin and RGopsin. Rhodopsin is a biological pigment found in the rods of the retina and is a G protein-coupled receptor (GPCR). It is extremely reactive to light. Opsins are a group of proteins made light sensitive via a chromophore sin photoreceptor cell of the retina. RGopsin targets a specific class of cones reacting to red/green light.

**Figure 10:**
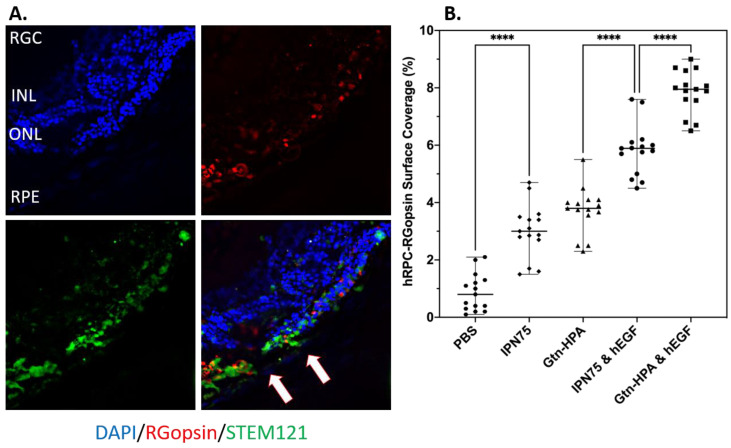
hRPCs survival, engraftment, and cone expression post-transplantation. (**A**) Immunohistochemistry of every 5th slide, for Gtn-HPA with hEGF group, stained with STEM121 (green), RG-opsin (red) and DAPI (blue). Scale bar: 200 µm. (**B**) Statistical analysis, using one-way ANOVA, followed by Pearson’s test, of the percentage of cell (expressing RG-opsin) surface coverage in injected groups. **** *p* < 0.0001.

To analyze the co-localization and engraftment of hRPCs expressing both human and either a rod/cone marker, we used an image processing algorithm described [11] previously which relies on Otsu’s method of thresholding. The analysis of RGopsin and STEM121 co-localization showed a high significant difference between groups, as seen in Figure 10B. Figure 10A shows an example of a field of view with hRPCs engrafted in the subretinal space and expressing both human marker (in green) and RGopsin (in red). By analyzing the surface coverage of the cells expressing both markers, we can compare not only the engraftment of hRPCs but the presence of specific subpopulation of hRPCs between different injection groups. By normalizing the surface coverage of double-stained hRPCs, we observed that cells encapsulated in Gtn-HPA with hEGF shows the highest amount of cone-hRPCs which is followed by the group of IPN75 with hEGF. Those two groups are significantly higher than both gel groups without hEGF. Finally, all groups with gel are significantly higher than cells injected in PBS. This is an important result which confirms the results we observed in the in vitro study: encapsulating hRPCs in Gtn-HPA with hEGF shows the highest controlled differentiation into cone-hRPCs in vitro and in vivo at longer time points. Of note is that a low amount of RGopsin cells was observed in the PBS groups, which is an agreement with the fact that hRPCs are mostly composed of rods.

**Figure 11:**
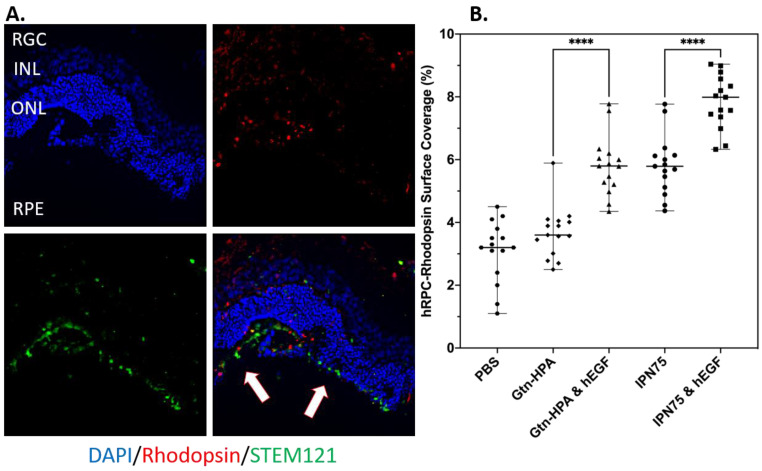
hRPCs survival, engraftment, and rod expression post-transplantation. (**A**) Immunohistochemistry for Gtn-HPA with hEGF group, stained with STEM121 (green), rhodopsin (red) and DAPI (blue). Scale bar: 200 µm. (**B**) Statistical analysis, using one-way ANOVA, followed by Pearson’s test, of the percentage of cell (expressing rhodopsin) surface coverage in injected groups. **** *p* < 0.0001.

The second marker we analyzed for co-localization is rhodopsin, which should be significantly higher due to the high presence of rods in hRPCs. As seen in Figure 11A, we were able to observe hRPCs engrafted in the subretinal space and expressing both human and rod marker in all groups, which allowed us to measure their surface coverage and statistically compare it. The results, showed in Figure 11B, are different from the results we obtained for RGopsin, with, for rhodopsin, the highest expressing group being cells encapsulated in IPN75 with hEGF. Of note is that IPN75 and Gtn-HPA with hEGF had a similar cell surface coverage being higher than Gtn-HPA. All gels groups were significantly higher than cells in PBS. This result confirms the in vitro phenotype assay which suggested that cells encapsulated in IPN75 with hEGF show the highest expression of rhodopsin.

### 2.5. Immunoresponse from Host for Encapsulated hRPCs in Different Hydrogels

**Figure 12:**
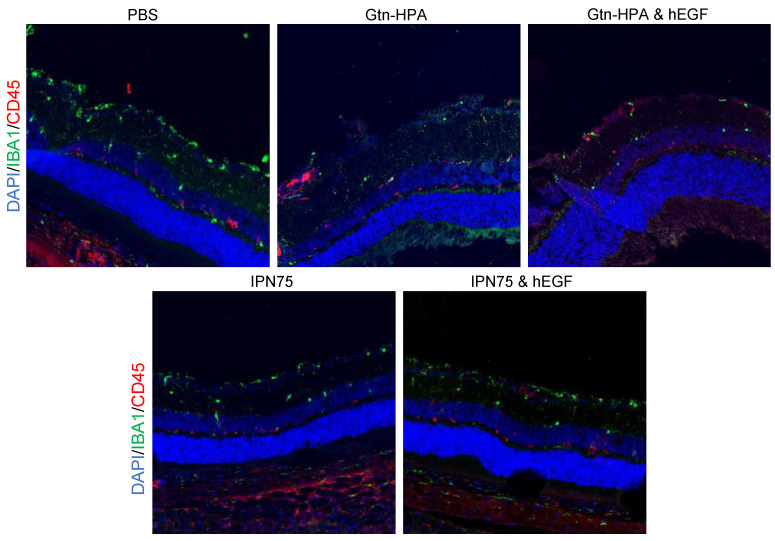
Immune response staining in xenografts. Images taken with fluorescence microscopy to identify leukocytes and immune cell marker expression. All images were taken at 63X magnification. Immunostaining of all groups (PBS, Gtn-HPA, Gtn-HPA and hEGF, IPN75, and IPN75 and hEGF) for CD45 (APC) identify leukocytes, with IBA1 (FITC) identifying microglial cells.

The short-term (3 days post-transplantation) immune reaction shows the injury caused by the injection was also analyzed. hRPCs injection (independent of the carrier) can trigger a reaction from the host as seen with leukocyte staining. In the control group (PBS), hRPCs are exposed to the invading leukocytes, especially in non-immunosuppressed animals, as seen in Figure 12.

## 3. Discussion

Overall, our findings in the in vivo experiment are critical as they confirm our results in the in vitro phenotype assay. Finally, we discovered that in order to enhance hRPC differentiation into cones Gtn-HPA with hEGF was the optimal hydrogel while to promote cell differentiation into rods IPN75 with hEGF was best suited. We also confirmed that these hydrogels can significantly enhance regeneration by acting as passive carrier in which a growth factor (hEGF in this case) can be added. This addition strongly enhanced the differentiation of hRPCs into either rods or cones.

In this study, we have analyzed the short- and long-term injection of hRPCs in rats. We injected hRPCs in PBS, Gtn-HPA, IPN75 with or without hEGF in the subretinal space of non-immunosuppressed rats of the Long–Evans strain. Cell suspensions (in PBS), injected into the subretinal space, showed the least viability and engraftment. In contrast, cells encapsulated in Gtn-HPA or IPN75 show evidence of better engraftment and cellular migration in the host retina. Cells were found migrating in the ONL and INL layers in the retina, while cells in PBS were clustered in the vitreous or RGC side of the retina. The percentage of cells that survived in the host retina was higher in Gtn-HPA grafts than in cell suspensions, suggesting Gtn-HPA was a superior cell carrier. Gtn-HPA is an biocompatible and biodegradable polymer [17] that forms a protective barrier for hRPCs, therefore protecting them from the migrating immune cells as seen from the leukocyte staining results. This short study aimed at proving the significant decrease in hRPCs presence, viability, and migration right after transplantation when injected in PBS compared to Gtn-HPA.

Indeed, it is known that hydrogen peroxide can greatly affect cell viability and proliferation even at low doses [18]. This suggests that we have found an optimal hydrogel to enhance the engraftment of hRPCs and increase the possible regeneration process. We were able to modify the stiffness but more importantly the gelation time of a pure hydrogel made of Gtn-HPA by mixing it with a different biodegradable and biocompatible hydrogel which possess a higher gel point (HA-Tyr). Therefore, we observed the creation of an IPN included in the class of in situ crosslinking hydrogels catalyzed with horseradish peroxidase that possess a high gelation time while maintaining cell viability and proliferation at an acceptable level.

The longer-term (3 weeks post-transplantation) in vivo experiment allowed us to compare our in vitro phenotype analysis with the possible phenotype expression of injected hRPCs in the subretinal space of Long–Evans rats. We were able to confirm our in vitro results by showing a similar trend for hRPCs encapsulated in different hydrogels with the addition of hEGF in the mix. Specifically, using Gtn-HPA with hEGF can enhance the differentiation of hRPCs into cones both in vitro and in vivo while IPN75 with hEGF promoted rods differentiation. These results are of importance due to the high heterogeneity of hRPCs [12,19] which are derived from fetal source and therefore can differentiate into any type of retinal cells (photoreceptors, RGC, bipolar, amacrine or horizontal cells). Being able to control their differentiation in vivo could lead to new applications focused on specific cell replacement, such as retinitis pigmentosa or rod-cone dystrophy.

To explain these in vivo results, we analyzed the stiffness of our different hydrogels. We measured that the addition of HA-Tyr in the hydrogel mix can linearly increase the shear and Young’s moduli of these gels [11]. Therefore, by increasing the stiffness of these hydrogels and using IPN75, we were able to control the differentiation of hRPCs into rods. The stiffness effect on stem cells in vitro and in vivo has been analyzed and observed in a other studies [14]. Here, we have considered stiffness as one of the many factors which can influence the fate of hRPCs. Other factors, such as oxygen content, chemical content, and ligand density, can also influence hRPC fate and should be analyzed as hydrogels to understand their effect on therapeutic cells.

The addition of hEGF in hydrogels can greatly improve the differentiation of hRPCs into rods or cones. hEGF is already being used in the defined media of many stem cell types, especially in the retina [20]. These hydrogels serve as a carrier in which one can include growth factors to promote cell viability, proliferation, and differentiation. In this study, we have evaluated both hEGF and hFGF which are used to culture hRPCs; however, many other growth factors could be used in order to drive the differentiation of hRPCs into rods, cones or other types of cells in the retina (such as GDNF).

As seen in past studies [21], the concentration of the catalyst (HRP) was shown to be optimal at 0.1 U/mL to enable encapsulated cells to thrive. In these studies, a live/dead assay on different types of stem cells (MSC, RPE, and ES) encapsulated in Gtn-HPA with varying concentrations of HRP was performed. The result show that a concentration of at least 0.1 U/mL was necessary to form a hydrogel. Concentration lower than 0.1 U/mL show a significantly low viability of cells (<20%) which is due to the presence of H_2_O_2_ cytotoxic to cells, not being used to create a gel as the concentration of HRP is too low. Upon using 0.1 U/mL a high viability was observed along with the formation of a hydrogel (80%) [21]. Increasing the concentration of HRP above 0.1 U/mL does not affect the viability of hRPCs. However, as shown in other studies [21], HRP might have a slight impact on cell phenotype and differentiation. As 0.1 U/mL has been shown to be completely used to catalyze the gelation of our hydrogels, these results suggest that it is the correct concentration to be used.

## 4. Conclusions

Together, these studies show the potential utility of specific hydrogel formulations to improve the delivery, survival, and differentiation of grafted cells in the retina. Modulating the stiffness of the gel directly impacts the diffusion rate, which in turn has downstream consequences for cell survival and maturation. In other words, encapsulating cells in 3D biomimicking hydrogels allows us to control the in vivo behavior of cells. These polymers may have use both in ocular therapeutics, as well as in other compartments of the body where stem or progenitor cells are delivered to promote healing or are used for cell replacement.

## 5. Materials and Methods

The effect of Gtn-HPA, HA-Tyr and IPN hydrogels on hRPCs was observed by designing multiple experiments with different culture conditions, as seen in Figure 1A. UC indicates base Ultraculture medium. To further analyze the impact and effect of these hydrogels (Gtn-HPA, HA-Tyr and IPNs) on hRPCs, we designed an experiment by encapsulating hRPCs in different formulations (modulating the content of gelatin in the mix) and adding different growth factors to promote cell growth and viability. For this purpose, we cultured hRPCs for 14 days, adding different growth factors (base medium deprived from growth factors, i.e., EGF, FGF or both), either in 2D culture in T75 flasks or encapsulated in hydrogels (Gtn-HPA, IPN25, IPN50, IPN75 and HA-Tyr). We analyzed hRPC viability, proliferation, and phenotype throughout the experiment at day 1, 4, 7, 11 and 14. Encapsulated hRPCs were then injected in the subretinal space of Long–Evans rats for a 3-week study. Rats were immunosuppressed with cyclosporin 3 days pre-transplantation (Figure 1B).

### 5.1. Human Retinal Progenitor Cell Culture

All human material work was performed with the approval of the Institutional Review Board of Harvard Medical School. hRPCs were isolated from human fetal neural retina at 16 weeks’ gestation as previously described [9]. Cells were cultured onto fibronectin (Akron)-coated flasks (surface 75 cm^2^, vented cap, sterile, Nunclon Delta) in Ultraculture medium (Lonza), supplemented with 10 ng/mL recombinant human basic FGF (Peprotech), 2 mM L-glutamine (Invitrogen), 20 ng/mL recombinant human EGF (Peprotech), and 0.4 mM Primocin (Invitrogen) in a low-oxygen incubator (37 °C, 5% O_2_ 5% CO_2_, 100% humidity) as a monolayer culture to achieve high density. Upon reaching 80% confluence, cells were passaged using 10X TrypZean (Sigma-Aldrich, St. Louis, MO, USA) and HBSS (Hank’s Balanced Salt Solution, no calcium, no magnesium, ThermoFisher, Waltham, MA, USA). Cell number and viability were estimated at each passage, using Trypan blue (Sigma-Aldrich) and a hemocytometer (Countess™ II FL Automated Cell Counter, Thermo Fischer Scientific). Cells were then re-plated onto a fibronectin coated T75 surface at a density of 15,000 cells/cm^2^ in the same medium. All work was performed with expanded hRPCs at passage 10.

### 5.2. Hydrogel Preparation and Crosslinking

In situ crosslinking of Gtn-HPA and HA-Tyr hydrogels was performed by an enzyme-catalyzed oxidation, as previously described [10], with horseradish peroxidase as a catalyzer and hydrogen peroxide as crosslinker. For the homopolymer hydrogels, horseradish peroxidase HRP (Wako USA) and hydrogen peroxide (H_2_O_2_) (Sigma-Aldrich) were added to solutions containing 2 wt% Gtn-HPA or HA-Tyr hydrogels to form final concentrations of 0.1 U/mL (HRP) and 1 mM (H_2_O_2_), respectively.

The concentrations of catalyst (HRP), crosslinker (H_2_O_2_), and polymer (Gtn-HPA and HA-Tyr) were chosen based on previous studies, which demonstrated their feasibility as a biocompatible and injectable hydrogel for subretinal and vitreous injection to enhance retinal regeneration [10,11].

Interpenetrating networks made from the mixing of Gtn-HPA and HA-Tyr at different proportions (IPN10, 25, 50, 75 and IPN90) were prepared by mixing the corresponding amounts of Gtn-HPA and HA-Tyr in a 2 wt% solution (e.g., where IPN75 corresponds to 75% of Gtn-HPA and 25% of HA-Tyr, both at 2 wt% solution). To create the IPN hydrogels, 0.1 U/mL of HRP and 1 mM of H_2_O_2_ were mixed into the solution. Hydrogels were formed after less than 5 min and incubated at 37 °C to reach stability. As both Gtn-HPA and HA-Tyr are catalyzed and crosslinked with the same molecules, a n IPN will be formed if no preferential crosslinking is observed (as explained previously [4]). Gelation time ranges from 30 to 90 s to and stability of the gels were obtained by incubation in a low-oxygen condition (37 C, 5% O_2_, 5% CO_2_) after 20 min.

### 5.3. Viability and Proliferation Assays

hRPCs at 5 × 10^5^/mL in PBS (Phosphate Buffer Saline, pH 7.4, ThermoFisher) or Ultraculture, with hEGF, with hFGF, defined media, or within 1 mL of hydrogels on top of fibronectin coated round cover slips glass (thickness 5 mm, diameter 1 cm, VWR) were incubated with 2.5 µM Calcein AM and 10 µM Ethidium homodimer-1 in PBS for 15 min at 37 °C, 5% CO_2_. Cells were then washed with PBS for 10 min three times at room temperature. Cover slips with cells were mounted on poly-l-lysine microscope slides (thickness 1 mm, L × W 75 × 25 mm, Thermo Scientific Shandon) with low viscosity slide mounting medium (Fisher Scientific) before imaging with an epifluorescence confocal microscope (Leica SP8, US). Cells in 15 randomly selected fields of view were counted under 20x objective lens magnification. hRPCs cultured in Gtn-HPA or HA-Tyr adopt a 3D configuration; therefore, a maximum projection over 300 um sample was applied to obtained live and dead imaging. Gtn-HPA and HA-Tyr are well-defined nanostructure hydrogels and hRPCs size have been studied (3 order of magnitude larger than the Gtn-HPA structure), we can assume that the images of the live/dead assay are indeed representing cells and not artefacts.

Proliferation assay was performed on the same samples by incubating cells in PBS, media, or hydrogels with 10 µM AlamarBlue (Bio-Rad) in PBS for 3 h at 37 ℃, 5/% CO_2_. Cells were then washed with PBS for 10 min three times at room temperature. Cytotoxicity and proliferation were measured with spectrophotometry (ThermoFisher). Absorbance at wavelengths of 570 and 600 nm after required incubation were measured. The curve of relative fluorescence units vs. drug concentration was generated with a 3-point correlation method.

### 5.4. Phenotype Assay with Flow Cytometry

hRPCs at 5 × 10^5^/mL in PBS or within 1 mL of hydrogels in 6-well plate (3.5 cm diameter, polystyrene, flat bottom, sterile, fisher scientific)] were maintained in PBS (replicating the in vivo conditions). At 1,3,7,11, and 14 days post-plating hydrogels were degraded using Collagenase IV and Hyaluronidase. 200 µL of phosphate-buffered saline (PBS) containing 1000 U/mL type IV collagenase (Invitrogen) was added to each sample before incubation at 37 °C on an orbital shaker at 150 rpm. Samples were collected 15 min later and centrifuged to isolate the cell pellet and analyze cell with flow cytometry or immunocytochemistry.

hRPCs (previously cultured in gel) were harvested and their phenotype was analyzed using flow cytometry with the MACSQuant flow cytometer (Miltenyi, San Diego, CA, USA). hRPCs, from different conditions (in media, in hydrogels, with or without hEGF/hFGF added) were collected and fixed with Perm/Fix buffer (BD Biosciences, Franklin Lakes, NJ, USA) at 4 °C for 15 min. Cells were then washed in wash buffer (BD Biosciences) and incubated, at room temperature, in blocking buffer (Pharmingen staining buffer with 2% goat serum) for 30 min. Blocked cells were seeded onto a flat bottom 96-well plate (treated, sterile, polystyrene, Thomas Scientific, Swedesboro, NJ, USA) and stained with conjugated primary antibodies (RG-opsin-APC, Recoverin-FITC, rhodopsin-APC, Oct4-APC, PAX6-APC, CMYC-APC) overnight at room temperature. Primary antibodies were diluted in 200 µL of antibody buffer (TBS, 0.3% Triton X-100 and 1% goat serum). Post-overnight incubation cells were washed three times for 15 min, and secondary antibodies (goat-derived anti-rabbit and anti-mouse, DAPI-VioBlue) were diluted 1:200 in antibody buffer (Jackson Immunoresearch Laboratory). Cells were incubated in secondary antibodies and left at room temperature for 3 h. Light scatter and fluorescence signals from each sample were measured using the MACSQuant (Miltenyi Biotech, Germany) flow cytometer (2 × 10^5^ events were recorded). The results were analyzed using the MACSQuantify software. For each primary antibody, the DAPI-positive single-cell population was gated. The ratio of positive cells in the gated population was estimated in comparison with blank and species-specific isotype control.

### 5.5. Oscillatory Rheology Measurement of Gel Stiffness

Oscillatory rheology was performed with a TA instruments AR-G2 rheometer using cone and plate geometry of 40 mm diameter and 2° angle. For each measurement, 200 µL of each sample (Gtn-HPA, IPN90, IPN75, IPN50, IPN25, IPN10 and HA-Tyr) at 2%wt/vol, containing 0.1 U/mL of HRP and varying concentrations of H_2_O_2_ (ranging from 0.8 to 1.3 mM) was applied to the bottom plate immediately after mixing. All hydrogels with a gelation time between 30 s and 3 min were still liquid when applied onto the bottom plate. The upper cone was lowered to a measurement gap of 51 µm. As soon as a layer of silicone oil was applied, to prevent evaporation, the rheometer was started. All measurements were taken at 37 °C in the oscillation mode with a constant strain of 1% and frequency of 1 Hz. G’ (storage modulus) and G’’ (loss modulus) were measured every 2 s. The final plateau value of G’ and time to reach this plateau were then recorded for each sample. Due to the fast gelation of all samples and time to stick the sample onto the bottom plate and the start of experiment, gel point was not measured with oscillatory rheology, this measurement can be seen in [11]. Evolution of the shear modulus G’, final plateau value, and critical time to reach this plateau were recorded for each sample. Each sample had *n* = 5 replicates to minimize the experimental effects of the rapid crosslinking time and oil application.

### 5.6. In Vivo Xenograft Study—Animals and Surgery

Twenty-five male rats of the Long–Evans strain (age 12 weeks, approximate weight 200 g), from Charles River (Wilmington, MA, USA), were used as recipients in the experiment. Transplantation was performed on cyclosporin immunosuppressed rats. Rats were sedated using 2–3% isoflurane (Abbott, Solna, Sweden) in combination with oxygen by placing the rats in the inhalation chamber, followed by intraperitoneal injection of ketamine (40–80 mg/kg) and xylazine (10 mg/kg) for anesthesia. Eyes were first anesthetized using topical ophthalmic proparacaine (0.5%) followed by Genteal to keep the lens moist during the surgery.

Recipient rats were injected in subretinal space with hRPCs encapsulated in composite suspension of hydrogels or single-cell injections (in PBS). A conjunctival incision and a small sclerotomy were performed using a fine disposable scalpel. Cells were injected into the subretinal space using a glass pipette (internal diameter, 150 µm) attached to a 10 µL Hamilton syringe via a polyethylene tubing. The hRPCs were injected into the retina bleb as a single-cell suspension in PBS (*n* = 5), encapsulated in 2%wt Gtn-HPA (n = 5), encapsulated in 2%wt Gtn-HPA with 20 ng/mL hEGF (*n* = 5), encapsulated in 2%wt IPN75 (*n* = 5), encapsulated in 2%wt IPN75 with 20 ng/mL hEGF (*n* = 5), as seen in Figure 2. All samples contained approximately 1 × 10^5^ cells and the injection volume were 2 µL for all replicates. Using a glass coverslip applied on the eye bleb presence was checked. Subretinal space injection was considered successful if a shiny bleb was seen under the dissection surgical microscope (Alcon Vitreoretinal, Constellation Vision System). Triple antibiotic (Bac/Neo/Poly) was given locally at the end of the surgery to prevent infection. The rats were then placed in their cages for 3 weeks. 100 mg/L of Cyclosporine was added to the water container of all cages and was changed every 3 days.

The research protocol was reviewed and approved by the Schepens Eye Research Institute Animal Facility and was in accordance with the Association for Research in Vision Ophthalmology Statement for the Use of Animals in Ophthalmic and Vision Research.

### 5.7. Control and Measured Outcomes

The control group consisted of healthy rats which had no surgery. The SHAM group consisted of rats which had only a minimal surgery consisting of poking the eyeball with a 31-gauge needle without injecting anything. This is performed to replicate the trauma of needle injection itself. These rats were sacrificed at the same time points as the experimental groups. Outcomes for this study were based on direct examination, image processing and machine learning analysis of retinal sections of injected rats. The measured outcomes were:

- % of cells engrafted expressing STEM121 (Human),
- % of cells engrafted expressing rhodopsin (Rods),
- % of cells engrafted expressing R/G opsin (Cones),
- % of cells engrafted expressing DAPI (Nuclei), and
- Position of engrafted in cells in retinal layer.

### 5.8. Tissue Processing

Three weeks post-transplantation, rats were sacrificed by CO_2_ suffocation for 5 min. Cervical dislocation was performed to certify death. Eyes were enucleated and placed in 4% paraformaldehyde for 24 h. Tissues were subsequently saturated with increased concentrations of sucrose (5%, 10%, 20%) containing Sorensen phosphate buffer. Eyes were left in 30% sucrose overnight or until dissection. The tissues were embedded in cryosection gelatin medium overnight and sectioned at 18 µm thickness on a cryostat. During the sectioning process, every 5th section was stained and examined by epifluorescence for hRPCs presence with STEM121-FITC (human cells marker), RGopsin-APC (cone marker) and DAPI-VioBlue (cell nuclei); every 6th section was stained with rhodopsin-APC (rod marker) instead of RG-opsin-APC.

### 5.9. Immunofluorescence Staining

Cryosections from Long–Evans rats left eye were fixed with 4% paraformaldehyde in 0.1 M PBS (Irvine Scientific) at room temperature for 20 min. These fixed cells and sections were blocked and permeabilized with a blocking solution (Tris-buffered saline (TBS), 0.3% Triton X-100 and 3% goat serum (Jackson Immunoresearch Laboratories, West Grove, PA, http://www.jacksonimmuno.com) for 15 min. Samples were then rinsed twice with 0.1 M TBS buffer for 15 min each time, mounted on polysine microscope slides and incubated with primary antibodies overnight at 4 °C (rhodopsin-APC, RG-opsin-APC, STEM121-FITC, DAPI-VioBlue) at concentrations determined in laboratory. Post-overnight incubation, samples were rinsed three times with TBS for 15 min. Secondary antibody (goat-derived anti-mouse and anti-rabbit, DAPI-VioBlue) staining was performed for 1 h at room temperature. Samples were then washed one last time with TBS before being mounted on poly-l-lysine microscope slide with low viscosity slide mounting medium. Digital images were obtained with an epifluorescence confocal microscope (Leica SP8) using 20× objective.

### 5.10. Image Processing and Analysis

Ten randomly selected images were taken for each sample. To statistically analyze the field of view, an image processing MATLAB code was used, as previously described [11]. For each image taken, the code calculates (with a tolerance of 0.01%) the surface covered by the cells of interest (in images taken with 20× magnification). The number of colored pixels (Green for FITC channel and Red for PE or APC channels) were counted and a percentage of cell surface coverage was created.

### 5.11. Statistical Analysis

All experiments were performed with *n* = 10–15. The power calculation was based on detecting a significant difference in the means between groups of 30% and 40% with a standard deviation of 15% and α = 0.05 and β = 0.20. Values were expressed as the mean +/− standard error mean (SEM) using GraphPad software. Analysis of variance (one-way and two-way ANOVA) followed by Tukey’s test and Student’s *t*-test were performed for statistical analysis for the IHC and flow analysis. Statistical significance was set at *p* < 0.01.

## Data Availability

The data that support the findings of this study are available from the Gilbert foundation database platform: https://nf.synapse.org/Explore/Studies/DetailsPage?studyId=syn21650484.
